# Cognitive behavioral therapy targeting persecutory delusions in chronic outpatients with schizophrenia: a randomized controlled trial on functional recovery and symptom severity

**DOI:** 10.3389/fmed.2026.1758031

**Published:** 2026-05-15

**Authors:** Jingjing Wang, Hailu Xu, Jin Yuan, Xiaoyan Zhang, Daoying Liu

**Affiliations:** 1 Department of Medical Humanities, Sias University, Xinzheng, Henan, China; 2 Department of VIP Ward, Affiliated Mental Health Center & Hangzhou Seventh People’s Hospital, Zhejiang University School of Medicine, Hangzhou, Zhejiang, China; 3 Department of the Third Psychiatry, Affiliated Mental Health Center & Hangzhou Seventh People's Hospital, Zhejiang University School of Medicine, Hangzhou, Zhejiang, China; 4 Department of Nursing, Affiliated Mental Health Center & Hangzhou Seventh People's Hospital, Zhejiang University School of Medicine, Hangzhou, Zhejiang, China

**Keywords:** anxiety, cognition, cognitive behavioral therapy, depression, quality of life, schizophrenia

## Abstract

**Background:**

Cognitive behavioral therapy (CBT) is a recommended psychosocial intervention for schizophrenia; however, its integrated application in routine clinical settings warrants further empirical evaluation.

**Aim:**

This study aimed to assess the effects of adjunctive, structured CBT on clinical symptoms, affective states, self-efficacy, and quality of life in hospitalized patients diagnosed with schizophrenia.

**Methods:**

In this randomized controlled study, 66 inpatients diagnosed with schizophrenia at the Affiliated Mental Health Center & Hangzhou Seventh People’s Hospital, Zhejiang University School of Medicine were recruited from August to October 2022. These participants were divided into two groups: a research group (RG, *n* = 33) and a control group (CG, *n* = 33). The CG received conventional pharmacotherapy, while the RG received pharmacotherapy in addition to a structured, individualized CBT intervention (once weekly, 60 min per session, for 4 weeks). Outcomes were assessed using the Positive and Negative Syndrome Scale (PANSS), Auditory Hallucinations Rating Scale (AHRS), Hamilton Anxiety Scale (HAMA), Hamilton Depression Scale (HAMD), General Self-Efficacy Scale (GSES), Brief Psychiatric Rating Scale (BPRS), and the World Health Organization Quality of Life-BREF (WHOQOL-BREF). Data were analyzed using *t*-tests, χ^2^ tests, and repeated-measures ANOVA where appropriate.

**Results:**

Compared to the CG, the RG showed significantly greater reductions in the PANSS total score (*p* < 0.05), AHRS score (*p* < 0.05), HAMA score (*p* < 0.05), HAMD score (*p* < 0.05), and BPRS score (*p* < 0.05). The RG also demonstrated significantly greater improvement in the GSES score (*p* < 0.05). In WHOQOL-BREF domains, the RG exhibited significant improvements in psychological, social relationship, and environmental scores, as well as in the total score (all *p* < 0.05), whereas the CG showed improvements only in psychological and social relationship scores.

**Conclusion:**

Adjunctive CBT was associated with significant clinical benefits in this inpatient sample, including reduced psychotic and affective symptoms, enhanced self-efficacy, and improved quality of life. These findings support the systematic integration of structured CBT into multidisciplinary inpatient care for schizophrenia. Future studies with larger, multi-center samples and longer follow-up periods are recommended to strengthen generalizability and evaluate the long-term effects of this treatment.

## Introduction

Schizophrenia is a serious and debilitating mental disorder characterized by diverse manifestations, including perceptual abnormalities, thought disturbances, and profound functional impairment, leading to a substantially reduced quality of life (1). While antipsychotic medications remain the pharmacological cornerstone, their limitations—such as side effects, residual symptoms (e.g., persistent hallucinations), and inadequate attention to psychosocial deficits—highlight the critical need for integrated, recovery-oriented interventions (2).

Cognitive behavioral therapy (CBT) has emerged as a leading evidence-based psychosocial intervention for schizophrenia. It is based on a conceptual framework that links dysfunctional beliefs, cognitive distortions, and emotional and behavioral outcomes (3). CBT for psychosis specifically targets symptom-related distress and functional impairment. Recent meta-analyses corroborate its efficacy in reducing the severity of positive symptoms (4). Moreover, a growing body of novel research highlights specific variables related to these findings: CBT demonstrates significant effects on the distress and frequency of auditory hallucinations, co-morbid anxiety and depression, and the enhancement of self-efficacy, which is a key predictor of functional recovery. Importantly, studies increasingly report CBT’s positive impact on quality of life (QoL), particularly in psychological and social domains, aligning with a holistic recovery model.

Despite this robust evidence base, significant research gaps persist, limiting clinical translation. First, the majority of evidence is derived from outpatient or specialized trial settings, and there is a paucity of controlled studies evaluating the efficacy of structured CBT within *routine inpatient care*, where patient acuity is higher and treatment delivery models differ. Second, although multidimensional assessment is advocated, there are few inpatient studies that concurrently evaluate CBT’s impact on a *comprehensive set of* var*iables*, including specific symptoms [e.g., hallucinations as measured by the Auditory Hallucinations Rating Scale (AHRS)], affective comorbidity, self-efficacy, and *domain-specific* QoL, within a single trial design. This limits the understanding of which recovery dimensions are most responsive to CBT in acute care settings. Third, the theoretical application of CBT to the inpatient context—focusing on rapid stabilization, distress tolerance, and engagement—remains under-explored in the literature, lacking a clear conceptual linkage between the intervention mechanism and the expected multidimensional outcomes in this population.

Therefore, this study is based on the cognitive model of psychosis and aims to address these translational and conceptual gaps. We hypothesize that supplementing standard pharmacotherapy with a structured, manualized CBT protocol tailored for inpatients will lead to superior outcomes across a targeted spectrum of variables, which include core psychotic symptoms, auditory hallucinations, affective symptoms, self-efficacy, and multidimensional QoL. By implementing a randomized controlled design in a routine inpatient setting, this research seeks to (1) provide specific evidence regarding the effectiveness of CBT in acute care, (2) clarify its profile of benefits across interconnected outcome domains, and (3) strengthen the theoretical rationale for integrating targeted cognitive-behavioral strategies into multidisciplinary inpatient treatment plans to foster holistic recovery.

## Methods

### Study design and rationale

This study used a prospective, randomized, single-blind, parallel-group controlled design. The design was selected to minimize bias and establish a causal inference regarding the additive effect of structured Cognitive Behavioral Therapy (CBT) to standard pharmacotherapy in an inpatient setting. The primary objective was to evaluate whether adjunctive CBT leads to greater improvement in a comprehensive set of outcome domains compared to pharmacotherapy alone over a 4-week inpatient period. The primary hypothesis was that the CBT group would demonstrate significantly greater reductions in overall psychotic symptom severity (PANSS total score) and auditory hallucination distress (AHRS). Secondary hypotheses posited that the CBT group would show superior improvements in affective symptoms (HAMA and HAMD), self-efficacy (GSES), general psychopathology (BPRS), and specific domains of quality of life (WHOQOL-BREF).

### Study setting

The study was conducted within the closed psychiatric inpatient units of the Affiliated Mental Health Center & Hangzhou Seventh People’s Hospital, Zhejiang University School of Medicine, a tertiary specialized mental health hospital. This setting represents a routine acute care environment where patients are admitted for stabilization of acute psychotic symptoms. The standard care protocol for all inpatients includes pharmacotherapy, nursing care, and generic supportive activities, but not protocol-driven individual psychotherapy.

### Participant recruitment and sampling technique

A consecutive sampling method was used to enhance representativeness and minimize selection bias. All newly admitted inpatients who met preliminary screening criteria during the recruitment window (August to October 2022) were sequentially invited to participate. This method ensured that the sample reflected the typical flow and acuity of patients in the setting.

### Inclusion and exclusion criteria

Inclusion criteria were: (1) age 18–78 years, (2) minimum educational level of junior high school (to ensure comprehension of psychotherapeutic materials and assessments), (3) primary diagnosis of schizophrenia confirmed by two attending psychiatrists according to the Chinese Classification of Mental Disorders, Third Edition (CCMD-3), and (4) maintained on a stable regimen of antipsychotic medication for at least 1 week prior to enrollment.

Exclusion criteria were as follows: (1) comorbid major mental disorders (e.g., substance use disorder, bipolar disorder, and severe intellectual disability); (2) severe impairment in emotional expression or interpersonal communication precluding engagement in therapy; (3) concurrent or recent (within 3 months) structured psychotherapy; (4) non-convulsive electroconvulsive therapy within the past month; and (5) significant, uncontrolled medical/neurological conditions.

### Randomization and allocation procedure

Eligible participants who provided written informed consent were randomly assigned to either the research group (RG) or the control group (CG). The randomization and selection process were as follows: A computer-generated random number sequence (block randomization with a block size of 4) was prepared by an independent statistician not involved in recruitment or assessment. This sequence defined the group allocation (RG or CG) for each consecutive participant ID. The allocations were concealed in sequentially numbered, opaque, sealed envelopes. After a participant completed the baseline assessment, the research coordinator opened the next envelope in sequence to reveal the allocation, thereby implementing the random selection into the study or control group. This process ensured allocation concealment and prevented selection bias.

### Sample size calculation

The sample size was determined *a priori* using G*Power software (version 3.1). Based on a projected medium effect size (Cohen’s *f* = 0.25) for the primary outcome (PANSS change) from prior similar studies (5), with an alpha of 0.05, power of 0.80, for a repeated-measures ANOVA (between-group factor), a minimum of 60 participants (30 per group) was required. To account for an estimated 10% attrition rate, 66 participants were enrolled (33 per group).

### Ethical considerations and informed consent

This study was conducted in accordance with the ethical principles of the Declaration of Helsinki. The research protocol, including all procedures involving human participants, was reviewed and approved by the Institutional Review Board (IRB) of the Affiliated Mental Health Center & Hangzhou Seventh People’s Hospital (Approval Reference Number: 2019012; Date of Approval: April 8th, 2019).

Written informed consent was obtained from all participating patients prior to their enrollment in the study. For patients who were critically ill or sedated and unable to provide consent at the time of admission, consent was initially obtained from their legally authorized representative (a first-degree relative). As soon as the patient’s condition improved and they regained decision-making capacity, they were fully informed about the study, and their personal written consent was subsequently obtained to confirm continued participation. The consent process was documented in the patient’s medical record.

Throughout the study, participants’ rights, safety, and wellbeing were prioritized. Participation was entirely voluntary, and participants or their representatives retained the right to withdraw from the study at any time without any impact on the standard of medical care they received. To ensure confidentiality, all collected data were de-identified at the point of entry. Each participant was assigned a unique study code number. All research documents, electronic databases, and biological samples were stored securely and were accessible only to the principal investigator and authorized study personnel. The de-identified dataset used for statistical analysis contained no personal identifiers.

## Methods

Patients in the CG received conventional pharmacological treatment. The type of medication was not restricted in this study, and the dosage was based on chlorpromazine equivalent doses.

Patients in the RG received individual CBT in addition to the conventional pharmacological treatment provided to the CG. The CBT intervention was conducted once per week, with each session lasting 60 min, for a continuous period of 1 month (four sessions in total).

### Practitioner qualifications and treatment oversight

CBT was delivered by licensed psychotherapists who held a master’s degree in clinical psychology or mental health and had completed at least 2 years of specialized training in CBT. All sessions were supervised by a senior CBT supervisor with over 10 years of clinical experience. Regular group case reviews were held weekly to ensure treatment fidelity and adherence to the protocol.

### Detailed CBT protocol

The CBT intervention followed a structured, phase-based approach, as outlined in the Table 1.

**Table 1:** Cognitive behavioral therapy protocol: phases, objectives, key techniques/components, and session frequency and duration.

Phase (week)	Objectives	Key techniques/Components	Session frequency and duration
Phase 1 (week 1)	Establish rapport, collect medical and psychosocial history, and introduce self-monitoring of thoughts and behaviors.	Clinical interview, psychological assessment, therapeutic alliance building, and thought records.	1 session, 60 min
Phase 2 (weeks 2–3)	Identify and modify distorted cognitions; develop coping skills for emotion and behavior regulation.	Psychoeducation, relaxation training, guided self-instruction, cognitive restructuring, behavioral experiments.	1 session/week, 60 min each
Phase 3 (week 4)	Consolidate skills and promote transfer to daily life; address medication-related misconceptions to enhance adherence.	Skill generalization planning, relapse prevention, medication education, homework review.	1 session, 60 min

Throughout the intervention, therapists guided patients to: (1) recognize the relationship between maladaptive emotions and behaviors, (2) practice managing automatic thoughts and modifying irrational beliefs, (3) apply relaxation and self-guidance techniques in real-life contexts to improve social adaptation, and (4) correct misconceptions about medication and its side effects through discussion, thereby reducing fear and encouraging recovery.

## Outcome measures

All outcome assessments were conducted by research psychiatrists who were trained to standardize rating procedures and were blinded to participant allocation. The Chinese-language versions of all instruments were used, which have undergone standard translation, back-translation, and cultural adaptation procedures, and have established reliability and validity in Chinese psychiatric populations.

### Primary outcomes

Psychiatric symptom severity was assessed using the Positive and Negative Syndrome Scale (PANSS) (6). The total score (range 30–210) and subscale scores for positive, negative, and general psychopathology symptoms were analyzed. A reduction in total PANSS score is indicative of clinical improvement.

Auditory hallucination severity was specifically measured using the Auditory Hallucinations Rating Scale (AHRS) (7), which provides a total score reflecting the frequency, loudness, content, and associated distress of hallucinations.

### Secondary outcomes

Depressive and anxiety symptoms were evaluated using the Hamilton Depression Rating Scale (HAMD) (8) and Hamilton Anxiety Scale (HAMA) (9), respectively. Higher scores indicate greater symptom severity.

General psychopathology and treatment response were assessed with the 18-item Brief Psychiatric Rating Scale (BPRS) (10). Treatment response was categorized as a ≥ 50% reduction in BPRS total score from baseline, with non-response defined as a < 25% reduction.

Patient-reported outcomes included:

Perceived Self-Efficacy: Measured using the 10-item General Self-Efficacy Scale (GSES) (11). Higher scores reflect a stronger belief in one’s ability to manage challenges.
Health-Related Quality of Life: Assessed via the World Health Organization Quality of Life-brief (WHOQOL-BREF) (12), which yields domain scores (Physical, Psychological, Social, Environmental) on a 0–100 scale, with higher scores denoting better quality of life.

### Statistical analysis

Statistical analysis was performed using SPSS 19.0 software. Measurement data were expressed as M ± SD and compared using an independent samples *t*-test. Between-group comparisons of symptom changes before and after the intervention were analyzed using repeated-measures ANOVA. Count data were expressed as rates (percentages) and compared using the χ2 test. A two-tailed *p*-value of <0.05 was considered statistically significant.

## Results

### General data

There were no statistical differences in sex ratio, age, and past medical history between the two groups (*p* > 0.05, Table 2), indicating that the general data of both groups were comparable.

**Table 2:** General data of patients in two groups.

Groups	Cases	Gender (Male/Female)	Age (years)	Course of disease (months)
Control group	33	18/15	46.34 ± 6.38	32.46 ± 18.5
Research group	33	17/16	45.64 ± 6.46	31.43 ± 18.7
χ^2^/t		0.060	0.442	0.224
*P*		0.805	0.659	0.822

### Changes in PANSS scores

**Figure 1:**
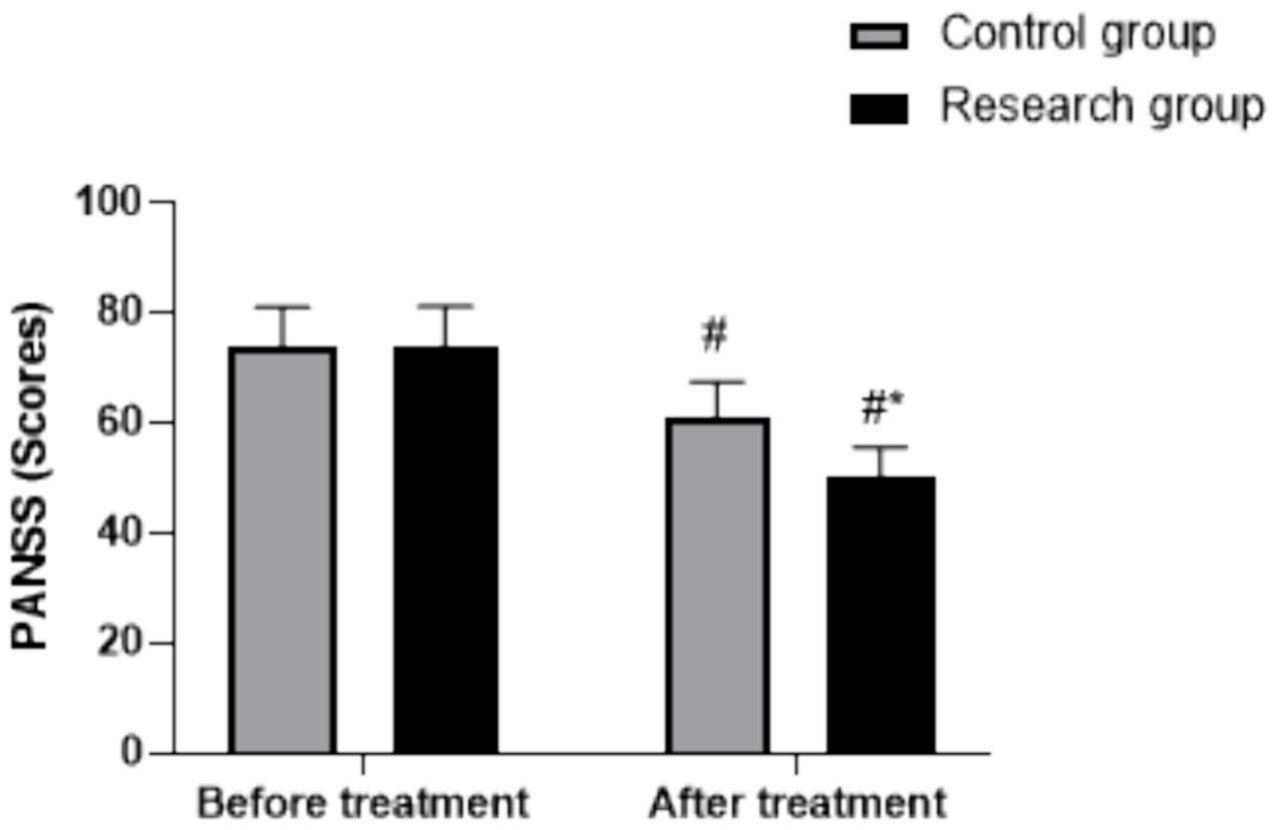
PANSS scores in both groups. ^#^ *p* < 0.05, compared to before treatment, * *p* < 0.05, compared to CG. PANSS, Positive and Negative Syndrome Scale.

A repeated measures ANOVA revealed a significant main effect of time (*F* (1, 118) = 212.54, *p* < 0.05) and a significant main effect of group (*F* (1, 118) = 35.67, *p* < 0.05). Furthermore, a significant group × time interaction effect was observed (*F* (1, 118) = 28.93, *p* < 0.05). Before treatment, there was no significant difference in PANSS total scores between the two groups (RG: 73.69 ± 7.42, CG: 73.67 ± 7.39, *p* > 0.05). After treatment, both groups showed a reduction in PANSS scores (*p* < 0.05, 95% CI: 15.62–20.20). However, the reduction was significantly greater in the RG (post-treatment: 50.29 ± 5.31) than in the CG (post-treatment: 61.25 ± 6.23) (*p* < 0.05, 95% CI: −7.759–−3.181), as shown in Figure 1.

### Changes in AHRS scores

**Figure 2:**
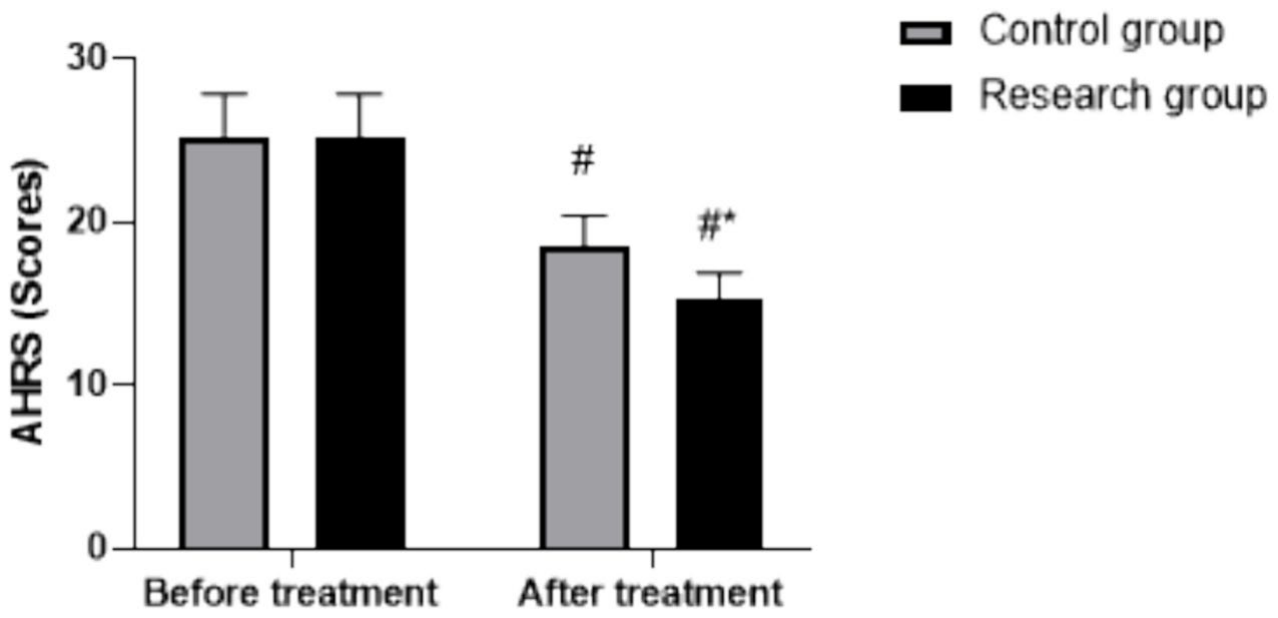
AHRS scores in both groups. # *p* < 0.05, compared to before treatment, * *p* < 0.05, compared to CG. AHRS, Auditory Hallucinations Rating Scale.

Repeated measures analysis indicated a significant main effect of time (*F* (1, 118) = 185.23, *p* < 0.05) and group (*F* (1, 118) = 22.14, *p* < 0.05), with a significant group × time interaction (*F* (1, 118) = 19.76, *p* < 0.05). Baseline AHRS scores did not differ between groups (RG: 25.23 ± 2.63, CG: 25.31 ± 2.58, *p* > 0.05). Post-treatment, AHRS scores decreased in both groups (*p* < 0.05, 95% CI: 7.630–9.170), with the RG showing a significantly greater reduction (post-treatment: 15.26 ± 1.65) than the CG (post-treatment: 18.48 ± 1.92) (*p* < 0.05, 95% CI:-2.420–−0.880), as shown in Figure 2.

### Changes in HAMA and HAMD scores

**Figure 3:**
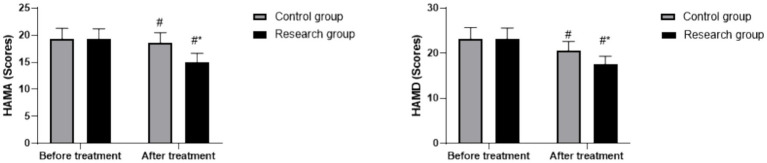
HAMA and HAMD scores in both groups. ^#^ *p* < 0.05, compared to before treatment, * *p* < 0.05, compared to CG. HAMA, Hamilton Anxiety Scale; HAMD, Hamilton Depression Scale.

For HAMA scores, a significant main effect of time (*F* (1, 118) = 91.45, *p* < 0.05), group (*F* (1, 118) = 19.05, *p* < 0.05), and group × time interaction (*F* (1, 118) = 15.89, *p* < 0.05) was found. For HAMD scores, significant main effects of time (*F* (1, 118) = 123.68, *p* < 0.05) and group (*F* (1, 118) = 17.32, *p* < 0.05), and a significant interaction (*F* (1, 118) = 12.74, *p* < 0.05) were observed. No baseline differences were observed in HAMA (RG: 19.32 ± 1.89, CG: 19.37 ± 1.92, *p* > 0.05) or HAMD scores (RG: 23.24 ± 2.41, CG: 23.25 ± 2.48, *p* > 0.05). Post-treatment, HAMA and HAMD scores decreased in both groups (*p* < 0.05, 95% CI: 1.863–3.117; *p* < 0.05, 95% CI: 3.376–4.884), with the RG showing a significantly greater reduction than the CG in HAMA (RG post-treatment: 15.26 ± 1.65, CG post-treatment: 18.65 ± 1.85; *p* < 0.05, 95% CI: −2.447–−1.193) or HAMD scores (RG post-treatment: 17.56 ± 1.80, CG post-treatment: 20.67 ± 1.99; *p* < 0.05, 95% CI: −2.314–−0.806), as shown in Figure 3.

### Changes in GSES scores

**Figure 4:**
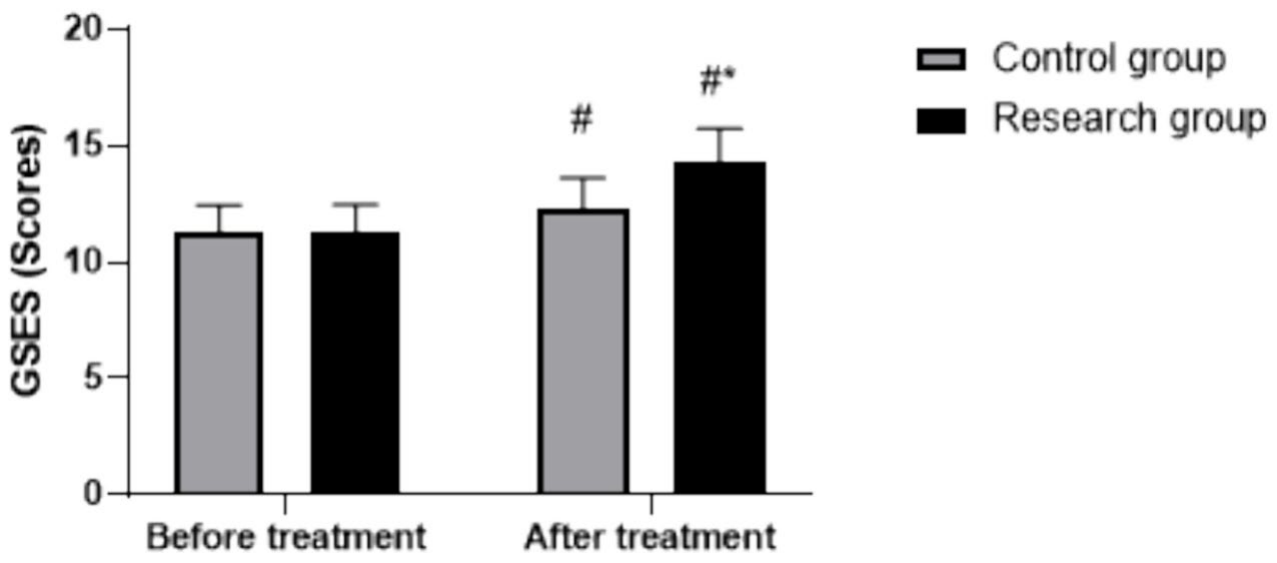
GSES scores in both groups. ^#^ *p* < 0.05, compared to before treatment, * *p* < 0.05, compared to CG. GSES, General Self-Efficacy Scale.

Repeated measures ANOVA showed significant main effects of time (*F* (1, 118) = 176.33, *p* < 0.05) and group (*F* (1, 118) = 25.81, *p* < 0.05), and a significant group × time interaction (*F* (1, 118) = 21.54, *p* < 0.05). Baseline GSES scores were comparable between groups (RG: 11.32 ± 1.15, CG: 11.28 ± 1.18, *p* > 0.05). Following treatment, GSES scores increased in both groups (*p* < 0.05, 95% CI: −2.457–−1.583), with the RG demonstrating a significantly greater increase (post-treatment: 14.28 ± 1.46) relative to the CG (post-treatment: 12.36 ± 1.26) (*p* < 0.05, 95% CI: 0.543–1.417), as shown in Figure 4.

### Changes in BPRS scores

**Figure 5:**
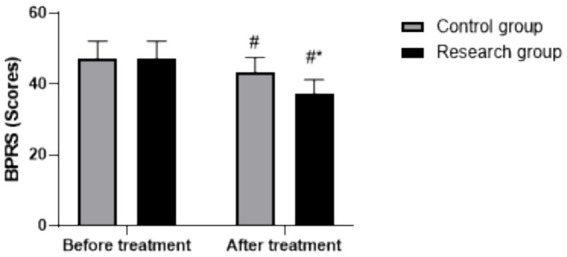
BPRS scores in both groups. ^#^ *p* < 0.05, compared to before treatment, * *p* < 0.05, compared to CG. BPRS, Brief Psychiatric Rating Scale.

The analysis revealed significant main effects of time (*F* (1, 118) = 145.28, *p* < 0.05) and group (*F* (1, 118) = 18.72, *p* < 0.05), as well as a significant group × time interaction (*F* (1, 118) = 16.39, *p* < 0.05). Before treatment, BPRS scores did not differ significantly (RG: 47.35 ± 4.85, CG: 47.36 ± 4.79, *p* > 0.05). After treatment, BPRS scores decreased in both groups (*p* < 0.05, 95% CI: 5.438–8.512), with the RG demonstrating a significantly greater decrease (post-treatment: 37.48 ± 3.82) relative to the CG (post-treatment: 43.28 ± 4.31) (*p* < 0.05, 95% CI: −4.442–−1.368), as shown in Figure 5.

### WHOQOL-BREF scores in both groups

**Figure 6:**
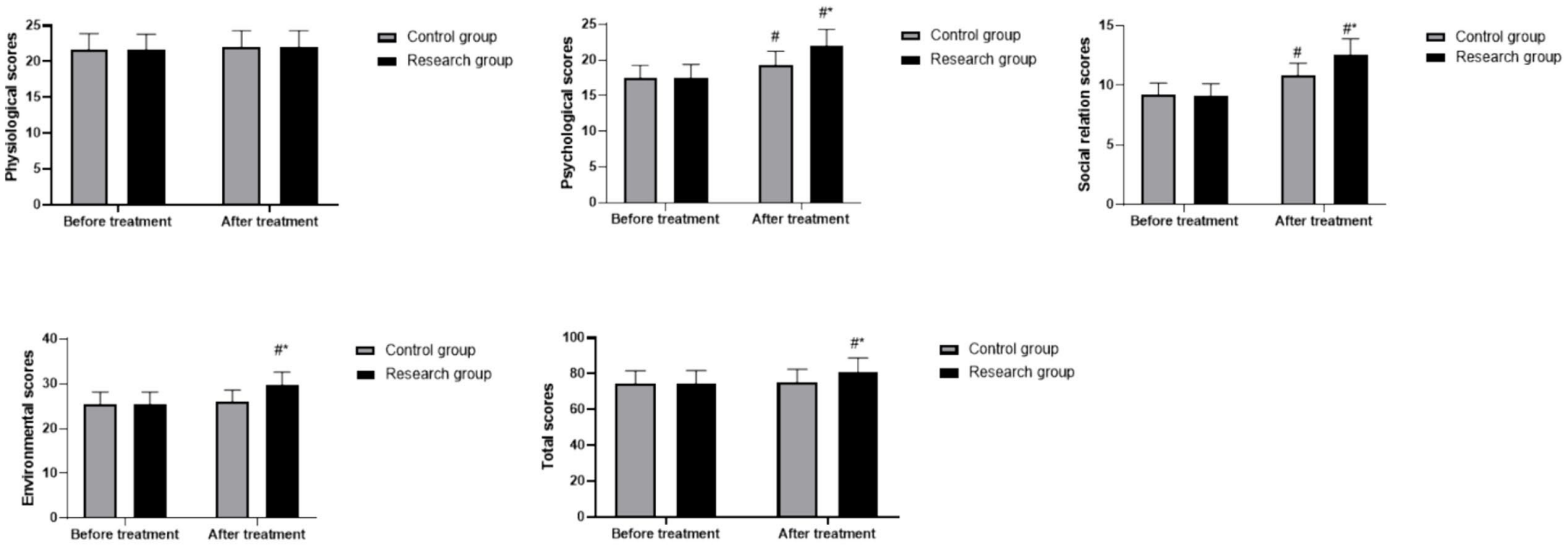
WHOQOL-BREF scores in both groups. ^#^ *p* < 0.05, compared to before treatment, * *p* < 0.05, compared to CG. WHOQOL-BREF, World Health Organization Quality of Life-brief.

Repeated measures ANOVA demonstrated significant main effects of time (*F* (1, 118) = 65.42, *p* < 0.05) and group (*F* (1, 118) = 15.38, *p* < 0.05), and a significant group × time interaction (*F* (1, 118) = 12.17, *p* < 0.05) for the total score. At baseline, all WHOQOL-BREF domain scores were similar between groups (all *p* > 0.05). In the CG, only the psychological domain (19.32 ± 1.87, *p* < 0.05) and social relationships domain (10.83 ± 1.03, *p* < 0.05) improved significantly after treatment. In the RG, significant improvements were observed in the psychological domain (post-treatment: 22.06 ± 2.23), social relationships domain (post-treatment: 12.62 ± 1.30), environmental domain (post-treatment: 29.69 ± 2.91), and total score (post-treatment: 80.79 ± 8.06) after treatment (all *p* < 0.05). Between-group comparisons indicated that the RG had significantly higher post-treatment scores than the CG in the psychological domain (*p* < 0.05, 95% CI: 0.743–2.077), social relationships domain (*p* < 0.05, 95% CI: 0.509–1.251), environmental domain (*p* < 0.05, 95% CI: 0.854–2.706), and total score (*p* < 0.05, 95% CI: 0.289–5.501) (Figure 6).

## Discussion

This study evaluated the effects of adjunctive CBT on patients with schizophrenia receiving conventional pharmacotherapy. The results demonstrated that, compared to pharmacotherapy alone, the combined intervention led to greater reductions in psychotic symptoms (PANSS), auditory hallucination severity (AHRS), and levels of anxiety (HAMA) and depression (HAMD). Furthermore, significant improvements were observed in self-efficacy (GSES), overall treatment response (BPRS), and quality of life (WHOQOL-BREF).

Cognitive impairment is a core feature of schizophrenia and is closely linked to poor psychosocial functioning (13). The improvements in self-efficacy (GSES) and patient-reported quality of life (WHOQOL-BREF) observed in our study are clinically significant outcomes that may reflect improved functional coping skills.

CBT may facilitate this improvement by helping patients in identifying and restructuring distorted automatic thoughts and dysfunctional beliefs. This process is well-supported by extensive literature (14). In this study, the reduction in positive symptoms, particularly auditory hallucinations (AHRS), may be attributed to CBT’s role in modifying catastrophic misinterpretations of internal experiences and developing alternative coping strategies. Similarly, reductions in negative symptoms (PANSS negative subscale) and improvements in general psychopathology could be linked to addressing defeatist beliefs and promoting behavioral activation, thereby reducing social withdrawal and feelings of amotivation (15). This cognitive-behavioral restructuring may help break the cycle of symptom distress, avoidance, and low mood, contributing to the observed reductions in comorbid anxiety and depression. Consistently, meta-analytic evidence supports the effectiveness of CBT in reducing symptoms and improving functional outcomes in individuals with schizophrenia (16).

From a neurobiological perspective, emerging evidence suggests that CBT may enhance cognitive function in schizophrenia by promoting neuroplasticity in the prefrontal regions involved in cognitive control and emotion regulation (17). This potential mechanism could provide a theoretical basis for improved top-down cognitive control over symptoms and affective states that may underpin gains in self-efficacy and clinical outcomes. The improvements in patient-reported quality of life align with findings from other trials (18), highlighting the value of targeting subjective recovery alongside symptom remission.

### Limitations and future directions

Several limitations of this study should be acknowledged. First, the sample size was relatively small and drawn from a single inpatient setting, which may limit the statistical power and the generalizability of the findings. Second, the absence of long-term follow-up data prevents conclusions regarding the durability of treatment effects beyond the immediate post-intervention period. Third, and as critically noted, while validated scales were used, the study did not include objective performance-based measures of functional capacity (e.g., UCSD Performance-Based Skills Assessment) or neurocognitive tests. This limits our ability to assess the intervention’s impact on objective functional skills and core cognitive domains and to elucidate precise mechanisms of action. Fourth, the CBT protocol, though structured, was delivered by therapists with varying levels of experience; future studies would benefit from systematic fidelity monitoring and detailed reporting of therapist training and supervision processes.

Based on these findings and limitations, we propose the following evidence-based recommendations:

For clinical practice: Structured, short-term CBT should be considered a valuable adjunct to pharmacotherapy within multidisciplinary inpatient psychiatric care, specifically targeting symptom distress, maladaptive coping, and subjective quality of life. Mental health nursing staff can play a pivotal role in reinforcing CBT principles during daily patient interactions, supporting behavioral activation, and monitoring changes in self-efficacy and mood.
For future research: High-quality, multi-center RCTs with larger samples and longer follow-up are needed. Future studies must integrate objective neurocognitive batteries and functional performance measures alongside self-report and clinical ratings to disentangle the effects of CBT on subjective appraisal versus objective cognitive/functional change. Implementation science frameworks should be used to optimize training, fidelity, and real-world delivery in diverse settings.
For education: Training curricula for psychiatrists and psychiatric-mental health nurses should emphasize the principles and techniques of evidence-based psychosocial interventions such as CBT. Enhancing clinicians’ competencies in delivering and supporting such interventions is essential for translating research findings into routine, integrated care.

## Conclusion

In summary, for the patients with schizophrenia in this study, adjunctive CBT demonstrated significant benefits when combined with conventional pharmacotherapy. The intervention was associated with reductions in core psychotic and affective symptoms, alongside improvements in self-efficacy and patient-reported quality of life. These outcomes represent key aspects of personal recovery. These findings support the value of integrating structured, short-term CBT into the current treatment framework for schizophrenia in similar clinical settings. Further research, incorporating objective functional and cognitive measures, is warranted to confirm and extend these results and to evaluate the long-term sustainability of the benefits.

## Data Availability

The original contributions presented in the study are included in the article/supplementary material, further inquiries can be directed to the corresponding author.

## References

[1] McCutcheon RA Reis Marques T Howes OD. Schizophrenia-an overview. JAMA Psychiatry. (2020) 77:201–10. doi: 10.1001/jamapsychiatry.2019.3360, 31664453

[2] Leucht S Bauer S Siafis S Hamza T Wu H Schneider-Thoma J . Examination of dosing of antipsychotic drugs for relapse prevention in patients with stable schizophrenia: a Meta-analysis. JAMA Psychiatry. (2021) 78:1238–48. doi: 10.1001/jamapsychiatry.2021.2130, 34406325 PMC8374744

[3] Roberts MT Shokraneh F Sun Y Groom M Adams CE. Classification of psychotherapy interventions for people with schizophrenia: development of the Nottingham classification of psychotherapies. Evid Based Ment Health. (2021) 24:62–9. doi: 10.1136/ebmental-2020-300151, 33355248 PMC10231480

[4] Reddy LF Glynn SM McGovern JE Sugar CA Reavis EA Green MF. A novel psychosocial intervention for motivational negative symptoms in schizophrenia: combined motivational interviewing and CBT. Am J Psychiatry. (2023) 180:367–76. doi: 10.1176/appi.ajp.20220243, 36891649

[5] Yan J Li K He Q Xiong J. Application of cognitive behavioural therapy combined with aripiprazole in the treatment of schizophrenia: a randomised controlled trial. Acta Neuropsychiatr. (2025) 37:e57. doi: 10.1017/neu.2025.14, 40143555 PMC13130281

[6] Buizza C Strozza C Sbravati G de Girolamo G Ferrari C Iozzino L . Positive and negative syndrome scale in forensic patients with schizophrenia spectrum disorders: a systematic review and meta-analysis. Ann General Psychiatry. (2022) 21:36. doi: 10.1186/s12991-022-00413-2, 36088451 PMC9463849

[7] Lindenmayer JP Kulsa MKC Sultana T Kaur A Yang R Ljuri I . Transcranial direct-current stimulation in ultra-treatment-resistant schizophrenia. Brain Stimul. (2019) 12:54–61. doi: 10.1016/j.brs.2018.10.002, 30316742

[8] Lu W Wang H Lin Y Li L. Psychological status of medical workforce during the COVID-19 pandemic: a cross-sectional study. Psychiatry Res. (2020) 288:112936. doi: 10.1016/j.psychres.2020.112936, 32276196 PMC7195354

[9] Zhang W Yan Y Wu Y Yang H Zhu P Yan F . Medicinal herbs for the treatment of anxiety: a systematic review and network meta-analysis. Pharmacol Res. (2022) 179:106204. doi: 10.1016/j.phrs.2022.106204, 35378276

[10] Hofmann AB Schmid HM Jabat M Brackmann N Noboa V Bobes J . Utility and validity of the brief psychiatric rating scale (BPRS) as a transdiagnostic scale. Psychiatry Res. (2022) 314:114659. doi: 10.1016/j.psychres.2022.114659, 35709637

[11] Weber-Rajek M Strączyńska A Strojek K Piekorz Z Pilarska B Podhorecka M . Assessment of the effectiveness of pelvic floor muscle training (PFMT) and extracorporeal magnetic innervation (ExMI) in treatment of stress urinary incontinence in women: a randomized controlled trial. Biomed Res Int. (2020) 2020:1019872. doi: 10.1155/2020/1019872, 32016111 PMC6988664

[12] Bahadur A Kumari P Mundhra R Ravi AK Chawla L Mahamood MM . Evaluate the effectiveness of enhanced recovery after surgery versus conventional approach in benign gynecological surgeries: a randomized controlled trial. Cureus. (2021) 13:e16527. doi: 10.7759/cureus.16527, 34430137 PMC8378282

[13] Hagi K Nosaka T Dickinson D Lindenmayer JP Lee J Friedman J . Association between cardiovascular risk factors and cognitive impairment in people with schizophrenia: a systematic review and Meta-analysis. JAMA Psychiatry. (2021) 78:510–8. doi: 10.1001/jamapsychiatry.2021.0015, 33656533 PMC7931134

[14] Guaiana G Abbatecola M Aali G Tarantino F Ebuenyi ID Lucarini V . Cognitive behavioural therapy (group) for schizophrenia. Cochrane Database Syst Rev. (2022) 7:Cd009608. doi: 10.1002/14651858.CD009608.pub2, 35866377 PMC9308944

[15] Xu F Xu S. Cognitive-behavioral therapy for negative symptoms of schizophrenia: a systematic review and meta-analysis. Medicine (Baltimore). (2024) 103:e39572. doi: 10.1097/md.0000000000039572, 39252302 PMC12431765

[16] Hong Y Chen Y Bai Y Tan W. Cognitive-behavioral therapy for the improvement of negative symptoms and functioning in schizophrenia: a systematic review and meta-analysis of randomized controlled trials. PLoS One. (2025) 20:e0324685. doi: 10.1371/journal.pone.0324685, 40392926 PMC12091889

[17] König P Zwiky E Küttner A Uhlig M Redlich R. Brain functional effects of cognitive behavioral therapy for depression: a systematic review of task-based fMRI studies. J Affect Disord. (2025) 368:872–87. doi: 10.1016/j.jad.2024.09.084, 39299583

[18] Petkari E Nikolaou E Oberleiter S Priebe S Pietschnig J. Which psychological interventions improve quality of life in patients with schizophrenia-spectrum disorders? A meta-analysis of randomized controlled trials. Psychol Med. (2024) 54:221–44. doi: 10.1017/s0033291723003070, 37859606

